# Association of *eNOS* and *HSP70* gene polymorphisms with glaucoma in Pakistani cohorts

**Published:** 2010-01-11

**Authors:** Humaira Ayub, Muhammad Imran Khan, Shazia Micheal, Farah Akhtar, Muhammad Ajmal, Sobia Shafique, Syeda Hafiza Benish Ali, Anneke I. den Hollander, Asifa Ahmed, Raheel Qamar

**Affiliations:** 1 Department of Biosciences, COMSATS Institute of Information Technology, Islamabad, Pakistan; 2 Department of Human Genetics, Radboud University Nijmegen Medical Centre, Nijmegen, The Netherlands; 3 Al-Shifa Trust Eye Hospital Rawalpindi, Pakistan; 4 Shifa College of Medicine, Islamabad, Pakistan; 5 Department of Ophthalmology, Radboud University Nijmegen Medical Centre, Nijmegen, The Netherlands

## Abstract

**Purpose:**

To investigate the involvement of stress-regulating genes, endothelial nitric oxide synthase (*eNOS*) and heat shock protein 70 (*HSP70*) with primary open angle glaucoma (POAG) and primary closed angle glaucoma (PCAG).

**Methods:**

POAG and PCAG patients recruited from different areas of Pakistan were diagnosed on the basis of clinical history, raised intraocular pressure (IOP), cup-to-disc ratio (CDR) and visual field defects. Their blood was collected and genomic DNA was extracted from it, followed by PCR amplification and VNTR typing of the *eNOS* gene, while the *HSP70* SNP was analyzed with PCR-RFLP. For both of the polymorphisms, the genotype distribution of the POAG and PCAG patients was compared with unaffected controls.

**Results:**

*HSP70* polymorphism was found to be significantly associated with PCAG (χ^2^=15.29 [p<0.001], OR=2.63 [95% CI=1.55–4.48]), with p<0.001 for the dominant model and OR=2.09 (95% CI=1.10–3.96) , with p<0.01 for the recessive model, but not with POAG (χ^2^=2.96 [p>0.05]). As opposed to this significant *eNOS* association*,* was seen with PCAG (χ^2^=6.33 [p<0.05], OR=2.09 [95% CI=1.12–3.89]), with p<0.01 for the dominant model, as well as with POAG (χ^2^=8.89 [p<0.05], OR=2.23 [95% CI=1.26–3.39]), with p<0.01 for dominant model. For the *eNOS *case, we found a significant association with the risk allele “a” for POAG patients (χ^2^=9.29 [p<0.01], OR=2.02 [95% CI=1.25–3.28, p=0.001]) and PCAG patients (χ^2^=7.59 [p<0.01], OR=1.99 [95% CI=1.18–3.37, p<0.01]). Similarly, in the *HSP70 *case, there was a significant association with the risk allele “C” for POAG patients (χ^2^=3.57 [p=0.05], OR=1.38 [95% CI=0.97–1.94, p<0.05]) and PCAG patients (χ^2^=18.32 (p<0.001), OR=2.16 [95% CI=1.49–3.13, p<0.001]).

**Conclusions:**

The intron 4 polymorphism of *eNOS* is associated with POAG, as well as PCAG, while the G+190C polymorphism in *HSP70* is associated with PCAG, but not with POAG in the Pakistani population.

## Introduction

Glaucoma is a multifactorial optic neuropathy characterized by apoptotic cell death of the retinal ganglion cells (RGCs) in the optic disc or retinal nerve fiber [1]. This irreversible retinal deterioration results in progressive visual field loss along with decreased contrast and color sensitivity [2]. Quigly et al. [3] have estimated that by the year 2010, approximately 60.5 million individuals will be affected by this disease, which causes bilateral blindness, and this number may rise to 79.6 million by the year 2020. Glaucoma is classified as a silent disease because patients usually do not have any signs and symptoms until the end stage, when considerable damage has been done to the eye [4, 5]. Increased intraocular pressure (IOP) is an established risk factor of the disease, along with old age, race and refractive error [6]. In addition to these factors, vascular [7], immunological [8] and neurotoxic [9] factors are also believed to cause glaucoma.

Wiggs et al. [10] point to genetics as an additional risk factor for the disease. Different strategies used to understand the genetic risk factors have helped to define the molecular events responsible for some Mendelian forms of the disease. In addition, some of the chromosomal locations of the genes that are likely to be involved in common forms of glaucoma have also been identified.

Galassi et al. [11] have shown that as a result of a variety of physiologic stresses, a highly conserved mechanism of gene regulation is activated. Stress or hypoxia in the tissues stimulates increased synthesis of Nitric oxide (NO), which results in an increased blood flow in the vessels of the tissue that helps to overcome the stress. Although several isoforms of nitric oxide synthase (NOS) have been reported in abundance in almost all layers of the retina, the circulating NO is synthesized solely in the vascular endothelium through the action of the endothelial nitric oxide synthase (eNOS) on the substrate L-arginine. Studies to identify the *eNOS* promoter element have revealed that many cis- and trans-acting factors together regulate the expression of the *eNOS* gene, and the level of *eNOS* expression, in turn, corresponds directly to the amount of NO in the blood [12]. Experiments have revealed that polymorphisms in the non-coding regions of *eNOS* may alter *eNOS* expression and thus cause a decrease in NO synthesis [13], which may predispose patients to hypertension, vasospasm and atherosclerosis.

In the vascular endothelium, an increase in the synthesis of NOS has been shown to be neuroprotective through the promotion of vasodilation and blood flow in the ocular tissue [11]. In contrast to previous models, which associated neuroprotection with higher eNOS activity, it has been observed that higher NO levels that correspond to an excess *eNOS* expression have pathological relevance and could be generally toxic. This pathogenicity model states that the excess NO freely diffuses to adjacent neurons and combines with O_2_**^-^** to form peroxynitrite anions (ONOO**^-^**), an extremely potent toxin which can set cell-death programs into motion, such as neuronal apoptosis [14]. An abundance of NOS has been found in the optic nerve head vessels of primary glaucoma patients, supporting the idea that the optic nerve damage in glaucoma can be related to *eNOS* overexpression [15].

In determining the risk factors associated with increased *eNOS* expression, it has been recognized that beside the many environmental and other physiologic factors, there is also a significant genetic contribution. One of the genetic risk factors that has been identified is the 27-bp variable number of tandem repeat (VNTR) polymorphism present in intron 4 of *eNOS*, which is thought to alter NO production and contribute to vascular deregulation [16]. In addition, it has been shown that vascular deregulation and abnormal IOP in POAG might together be responsible for causing optic nerve damage, and both of these factors cause a reduction in ocular perfusion [11]. No study has been conducted to define the relationship of the vascular deregulation and PCAG.

The mechanical stress resulting from elevated IOP, ischemia, excessive excitatory amino acids and excessive TNFα release increases stress in the optic nerve head [17]. Another mechanism elicited in response to stress is the recruitment of specific cellular proteins called “heat shock proteins” (HSPs), which have been shown to have a protective function [18], as the synthesis of the HSPs is upregulated in response to many forms of metabolic stress. These proteins are molecular chaperones that function in the proper refolding of the dysfunctional proteins and prevent protein aggregation, a mechanism that could be critical to the survival of RGCs; thus, a low expression of HSPs will abolish the defense mechanism against heat shock and stress in general [18]. Several investigators have shown that anti-HSP antibodies, through the induction of the apoptotic mechanism or the HSPs themselves via the stimulation of an immune response, may have pathogenic significance for patients with glaucomatous optic neuropathy [19].

The present study was conducted to determine whether *HSP70* and *eNOS* polymorphisms were associated with POAG and PCAG in the Pakistani population. We hope that our results will contribute toward a better understanding of the disease’s causative agents.

## Methods

This was a multicenter case-control study in which the patients and controls were randomly selected from different hospitals in the area. The participants included 166 unaffected controls, 159 POAG patients and 111 PCAG patients.

The patients were selected on the basis of their clinical history, cup–to-disc ratio (CDR) evaluation, visual field evaluation and elevated IOP, and categorized into POAG and PCAG groups based on gonioscopic findings. In addition, to rule out any ocular anomaly, the controls also underwent applanation tonometery, slit lamp examination, CDR measurement and visual field assessment. Blood samples from all of the individuals were collected by venipuncture after informed written consent was obtained, and genomic DNA was extracted from these blood samples through a conventional phenol chloroform method [20]. This study has been approved by the Departmental Ethics Committee and conforms to all of the norms of the Helsinki Declaration.

**Figure 1:**
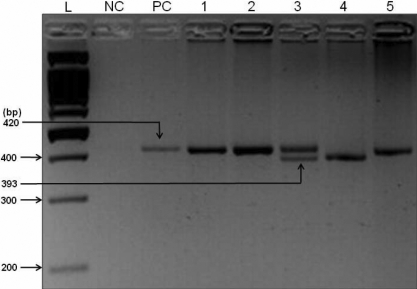
Amplification product of the 27-bp insertional variable number of tandem repeat polymorphism in intron 4 of *eNOS*. The amplified products were separated by electrophoresis on 4% agarose gel. Lane L, 100-bp DNA ladder; lane NC, negative control (no template DNA); lane PC, positive control; lane 1, 2 and 5, homozygous *eNOSb*/b genotype (420 bp fragment); lane 3, heterozygous, *eNOS*a/b genotype (420-bp and 393-bp fragments); and lane 4, homozygous a/a genotype (393-bp fragment).

The 27-bp insertional VNTR in intron 4 of *eNOS* was typed with the use of PCR amplification. The VNTR region was amplified using the Gene amp^®^ PCR system 2700 (ABI, Foster City, CA), with the help of the forward primer 5′-AGG CCC TAT GGT AGT GCC TTT-3′ and the reverse primer 5′TCT CTT AGT GCT GTG CTC AC-3′. Briefly, the protocol consisted of 35 cycles of DNA denaturation at 95°C for 1 min, primer annealing at 60°C for 45 s and chain extension at 72°C for 1 min, followed by a final extension cycle at 72°C for 5 min. The constituents of the reaction consisted of: 1.2 µM of each primer, 10 mM of dNTPs, 2 mM of MgCl_2,_ 1 U of Taq and 1× PCR buffer, along with 40–50 ng of DNA. The PCR products were electrophoresed on 4% agarose gels for 1 h and the bands were visualized under ultraviolet light (Figure 1). Two alleles were obtained when this region was amplified: “eNOS4a,” which was 393 bp long and consisted of four 27-bp repeating units, and “eNOS4b,” which was 420 bp long and consisted of five 27-bp repeating units.

**Figure 2:**
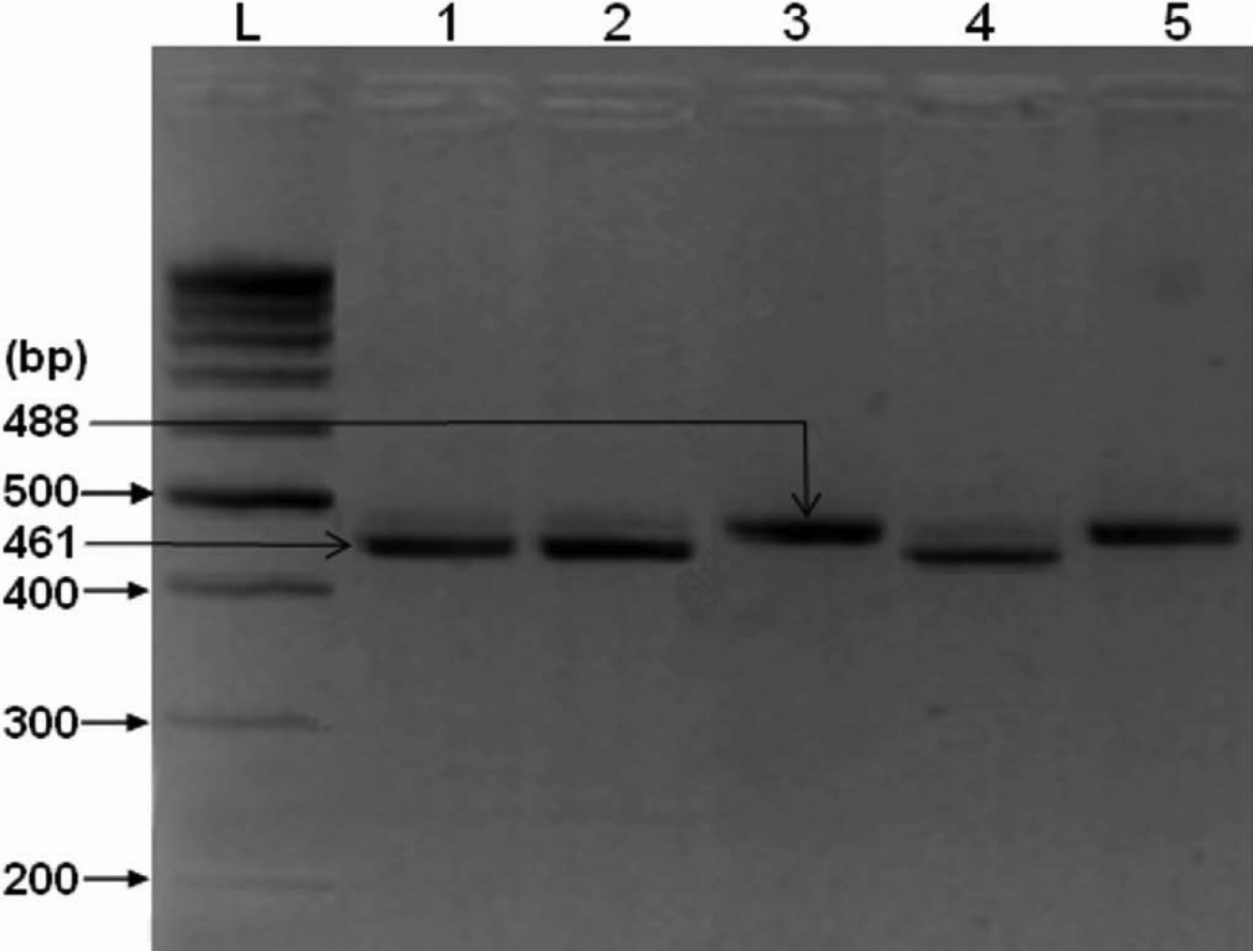
BrsB1 restriction enzyme-digested, PCR-amplified product of *HSP70* G+190C polymorphism (rs1043618). The digested products were separated by electrophoresis on 4% agarose gel. Lane L, 100-bp DNA ladder; lanes 1, 2 and 4, homozygous wild-type G allele (461 bp and 27 bp [not shown]); and lane 3 and 5, homozygous C risk allele (488 bp).

A 488-bp fragment encompassing the *HSP70* polymorphism (rs1043618) was PCR-amplified by using the forward and reverse primers, 5′-CGC CAT GGA GAC CAA CAC CC-3′and 5′-GCG GTT CCC TGC TCT CTG TC-3′, respectively. Each of the 35 cycles consisted of a DNA denaturation step at 95°C for 1 min followed by primer annealing at 60°C for 45 s and chain extension at 72°C for 1 min. The concentration of both primers was 0.04 µM, and the reaction mixture also contained 0.2 mM dNTPs, 1.5 mM MgCl_2_, 1 U Taq, 1× PCR buffer, and approx 90–100 ng of DNA. The PCR-amplified products (17 µl) were then digested overnight with BrsB1, as recommended by the manufacturer (Fermentas, Burlington, Ontario). After digestion, the restriction enzyme was inactivated at 65°C for 20 min, and the digestion products were electrophoretically separated on 4% agarose gel for 2 h and visualized under UV transillumination (Figure 2). The wild-type G allele was recognized based on the presence of a single BrsB1 restriction enzyme recognition site that, after digestion, yielded a 461-bp and a 27-bp fragment, while the C allele that lacks the restriction site remained uncut.

Genotype and allele frequencies in the unaffected controls and the patients (POAG and PCAG) were compared with the Pearson χ^2^ test. All of the data were analyzed by using SPSS (ver. 16; SPSS, Chicago, Il). The statistically significant differences were further analyzed using the statistical software Minitab (ver.15; Minitab, Chicago, Il). A p value of less than 0.05 was considered statistically significant.

## Results

Patients with POAG and PCAG and the controls were genotyped for the *eNOS4*a/b and *HSP70* G+190C polymorphisms. In the controls, the frequencies of the *eNOS4*b/b, *eNOS4*b/a and *eNOS4*a/a were 83.7%, 12.7%, and 3.6%, respectively, whereas in the POAG group, these values were 70%, 24% and 6%, respectively, showing a significant association of the disease with the polymorphism (χ^2^=8.89, p*<*0.05). In the PCAG group, 71%, 22% and 7%, respectively, were also found to be significantly associated with the disease phenotype (χ^2^=6.33, p*<*0.05; Table 1). The *eNOS4*b/a genotype was found to be significantly associated with POAG (χ^2^=6.92 [p*<*0.01]), as well as with PCAG (χ^2^=3.93 [p*<*0.05]). As opposed to this finding, the *eNOS4*b/b genotype was found to be at a significantly higher frequency in the control samples when compared with the POAG group (χ^2^=8.87, p*<*0.01) or the PCAG group (χ^2^=6.26, p=0.01; Table 1). In addition to the genotype associations, the *eNOS4*a allele frequency was found to be significantly associated with both POAG ([χ^2^=9.29, p*<*0.01], OR=2.02 [95%CI=1.25–3.28, p=0.001]) and PCAG ([χ^2^=7.59, p<0.01], OR=1.99 [95%CI=1.18–3.37, p<0.01]; Table 1).

**Table 1:** Genotype and allele frequencies of intron 4 polymorphism of *eNOS* in controls and patients.

**Genotype**	**Controls (n=166)**	**POAG (n=159)**	**p (χ^2^)**	**p (χ^2^)**	**Odds ratio (95% CI)**	**PCAG (n=111)**	**p (χ^2^)**	**p (χ^2^)**	**Odds ratio (95% CI)**
b/b	139 (83.7%)	111 (70%)	<0.05 (8.89)	<0.01 (8.87)	DM=2.23 (1.26–3.39) p<0.01	79 (71%)	<0.05 (6.33)	0.01 (6.26)	DM=2.09 (1.12–3.89) p<0.01
b/a	21 (12.7%)	38 (24%)		<0.01 (6.92)		24 (22%)		<0.05 (3.93)	
a/a	6 (3.6%)	10 (6%)		>0.05 (1.24)	RM=1.79 (0.58–5.69) p>0.05	8 (7%)		>0.05 (1.79)	RM=1.16 (0.40–3.31) p>0.05
**Allele**	**Controls**	**POAG**	**p (χ^2^)**		**Odds ratio (95% CI)**	**PCAG**	**p (χ^2^)**		**Odds ratio (95% CI)**
b	299 (90%)	260 (82%)	<0.01 (9.29)		2.02 (1.25–3.28) p=0.001	182 (82%)	<0.01 (7.59)		1.99 (1.18–3.37) p<0.01
a	33 (10%)	58 (18%)				40 (18%)			

DM=dominant model, RM=recessive model

The univariate logistic regression analysis revealed that the *eNOS4*a/b polymorphism was significantly associated with glaucoma (both POAG and PCAG), based on the dominant model of inheritance (for POAG, OR=2.23 [95% CI=1.26–3.39, p<0.01] and for PCAG, OR=2.09 [95% CI=1.12–3.89, p<0.01]), but not according to the recessive model (for POAG, OR=1.79 [95% CI=0.58–5.69, p>0.05] and for PCAG, OR=1.16 [95%CI=0.40–3.31, p>0.05]; Table 1).

To study the association of *HSP70* polymorphism G+190C with glaucoma, the POAG and PCAG patients, as well as the control samples, were genotyped. In the control group, the frequency of GG, GC and CC genotypes was 58%, 27% and 15%, respectively. For POAG patients, this distribution was 48%, 32% and 20%, respectively, with no significant association found for the genotype distribution in the patients, as compared to the controls (χ^2^=2.96, p>0.05). In PCAG patients, the frequency of the GG, GC and CC genotypes were 34%, 39% and 27%, respectively, and the genotype distribution was found to be significantly associated with the disease (χ^2^=15.29, p*<*0.001). The genotype CC was found to be significantly associated with PCAG (χ^2^=5.26, p=0.01), but not with POAG (χ^2^=1.12, p*>*0.05). In addition to the genotype distribution, the frequency of the G allele was found to be at a significantly lower frequency in both patient groups compared to in the control group, whereas the frequency of the C allele was high in the POAG (χ^2^=3.57, p=0.05, OR=1.38 [95% CI=0.97–1.94, p<0.05]) and PCAG groups (χ^2^=18.32, p*<*0.001, OR=2.16 [95% CI=1.49–3.13, p*<*0.001]; Table 2).

**Table 2:** Genotype and allele frequencies of G+190C polymorphism of *HSP70* in controls and patients.

**Genotype**	**Controls (n=166)**	**POAG (n=159)**	**p (χ^2^)**	**p (χ^2^)**	**Odds ratio (95% CI)**	**PCAG (n=111)**	**p (χ^2^)**	**p (χ^2^)**	**Odds ratio (95% CI)**
G/G	96 (58%)	77 (48%)	>0.05 (2.96)	>0.05 (2.88)	DM=1.44 (0.91–2.29) p>0.05	38 (34%)	<0.001 (15.29)	<0.001 (14.38)	DM=2.63 (1.55–4.48) p<0.001
G/C	45 (27%)	51 (32%)		>0.05 (0.96)		43 (39%)		<0.05 (4.15)	
C/C	25 (15%)	31 (20%)		>0.05 (1.12)	RM=1.37 (0.74–2.54) p>0.05	30 (27%)		0.01 (5.26)	RM=2.09 (1.10–3.96) p<0.01
**Allele**	**Controls**	**POAG**	**p (χ^2^)**		**Odds ratio (95% CI)**	**PCAG**	**p (χ^2^)**		**Odds ratio (95% CI)**
G	237 (71%)	205 (64%)	0.05 (3.57)		1.38 (0.97–1.94) p<0.05	119 (54%)	<0.001 (18.32)		2.16 (1.49–3.13) p<0.001
C	95 (29%)	113 (36%)				103 (46%)			

DM=dominant model, RM=recessive model

Univariate analysis of the *HSP70* G+190C polymorphism revealed that the polymorphism was not associated with POAG when the data was analyzed according to a dominant model (OR=1.44 [95% CI=0.91–2.29, p>0.05]) or a recessive model (OR=1.37 [95% CI=0.74–2.54, p*>*0.05]). However, an analysis of PCAG revealed the data to be significantly associated based on both the dominant (OR=2.63 [95% CI=1.55–4.48, p*<*0.001]) and recessive models (OR=2.09 [95% CI=1.10–3.96, p<0.01]).

## Discussion

Glaucoma is a disease leading to optic neuropathy and the second leading cause of blindness in the world. The slow, progressive loss of the retinal nerve ganglion cells in glaucoma results in peripheral vision loss. Glaucoma is a multifactorial disease and its exact etiology is still unknown. It has been reported that both genetic and environmental factors are significant in the progression of the disease. A multitude of recent genetic linkage studies have led to the identification of genetic loci, while genome wide search techniques have been used to identify single nucleotide polymorphisms, which have been observed to be associated with the disease.

In the present study, we provide data on two different polymorphisms of two genes, the 27-bp repeat *eNOS* VNTR polymorphism and the *HSP70* G+190C polymorphism. The *eNOS* and *HSP70* genes have been shown to elicit enhanced transcription levels of the respective mRNA when the body is under stress [18,21].

eNOS is involved in different processes, like neurotransmission, the regulation of vascular tone, vasodilatation and apoptosis. In addition, it also regulates blood flow to the ocular tissues and has been implicated in the pathogenesis of cardiovascular diseases and different neurodegenerative disorders, like diabetic retinopathy [22], glaucoma [23] and migraines [24]. In focusing on glaucoma, Liu and Neufeld [21] demonstrated that RGC degeneration in the glaucomatous optic nerve head of POAG patients clearly corresponds to excess plasma NO-mediated neurotoxicity. However, the authors did not conduct any genetic studies to find the susceptible loci.

Plasma NO levels are regulated by eNOS, thus any environmental or genetic risk factor that enhances *eNOS* expression would contribute to NO mediated toxicity. It has been established that the VNTR in the intron 4 of *eNOS* significantly influences the plasma NO levels. Functional studies have shown that the variant a allele (with four 27-bp repeats) in homozygous form is strongly correlated with increased plasma NO levels, which were twice as high as the level found in the b/b genotype (with five 27-bp repeats), indicating the *eNOS*’ underlying potential for excess NO-related pathogenicity [25].

It was hypothesized that the molecular mechanism of *eNOS* expression was regulated at the transcriptional levels by microRNAs, which can be derived from the intron during pre-mRNA splicing [26]. Based on this hypothesis, Zhang et al. [27] conducted experiments and established the role of intron 4 VNTR repeat with *eNOS* expression by identifying a 27-nucleotide microRNA sequence derived from the 27-nucleotide repeat within intron 4 of *eNOS*. This microRNA was shown to represses *eNOS* expression by interfering with the gene transcription efficiency. In addition, they demonstrated that this microRNA acted as an endogenous sequence-specific feedback regulatory molecule that reversibly inhibited this process and thus enabled the rapid turnover of eNOS molecules to minimize the damage associated with stress and hypoxia. The presence of the endogenous expression of the microRNA from intronic sequences was further demonstrated by the unexpected finding that the 5′ and 3′ ends of the 27-nucleotide repeat unit in intron 4 of *eNOS* had splice donor and acceptor sites (AG and GA) similar to the 5′ and 3′ bases at the exonic ends in the *eNOS* genomic sequence. This further supported the hypothesis that the intact microRNAs are produced when pre-mRNA splicing of *eNOS* occurs and that the amount of microRNA is determined by the number of repeats in intron 4 [27].

Zang et al. [27] also postulated that the amount of microRNA would vary from individual to individual, depending on the number of repeats at this position, which would account for the variations observed between the individuals and their expression of *eNOS* and its association with cardiovascular risk. Consequently, compared to the b allele with five 27-bp repeats, variant a allele with 4 repeats produced fewer microRNAs and hence, reduced transcriptional repression would occur, correlating with the increased plasma NO. Our current results are in line with the above results, as we found a significant association of the *eNOS4* a/b genotype with both forms of glaucoma in the Pakistani groups, which is probably a result of high plasma NO levels that are toxic to the optic nerve and ultimately cause its degeneration, a classic hallmark of glaucoma. Earlier studies conducted to establish a possible association between glaucoma and altered NOS expression focused mainly on POAG patients [11,12] and were able to show a positive correlation between this polymorphism and the disease, matching our results.

In the current work, we also studied PCAG as an impairment in the ocular blood flow that might lead to closure of the angle, which can be partially associated with altered *eNOS* expression and the resultant NO-mediated neurotoxicity. Our results indicate that there is indeed a correlation between PCAG and the polymorphism in the *eNOS* gene, as we found a significantly higher frequency of the variant *eNOS4*a allele in PCAG patients compared to the controls. Thus, in the Pakistani PCAG and POAG groups, the disease could be associated with increased NOS activity.

Our findings are consistent with the results of Sakai et al. [28], who found the T(−786)C polymorphism of *eNOS* to be a risk factor in patients developing non-arteritic anterior ischemic optic neuropathy (NAION) disease [28]. However, while studying glaucoma, Logan et al. [13] were unable to show a significant association between *eNOS* polymorphism T786C and VNTR repeat polymorphism and the disease. Similarly, Sena et al. [29] and Lin et al. [30] did not find any association between POAG and *eNOS* intron 4 VNTR. As opposed to this finding, however, Colombo et al. [31] have shown an association between Glu289Asp and T788C polymorphism of *eNOS* and cardiac myopathies, wherein patients who carried both polymorphisms had a higher risk of developing the disease. Polak et al. [32] studied the role of the NO system in glaucoma patients by giving POAG patients and normal healthy controls NG-monomethyl-L-arginine, which is an NOS inhibitor. They observed that glaucoma patients did not respond to the drug due to abnormal NOS activity, whereas healthy controls showed reduced choroidal and optic nerve head blood flow [32].

NO is responsible for maintaining arteries’ vasodilation, which keeps the ocular blood flow constant. When the endothelial function is deregulated, the blood supply to the tissue is altered and impaired blood flow damages the optic nerve and leads to the development of glaucomatous changes in the optic nerve, which then results in an increase in the CDR. In our study group, an increased CDR, as compared to the controls (C/D=0.29±0.11), was observed in the POAG group (C/D=0.75±0.24, p<0.001), as well as in the PCAG group (C/D=0.67±0.28, p<0.001). This increased CDR in both patient groups is evidence of excessive optic nerve damage, possibly as a result of NO activity. In addition to the differences in the CDR between the controls and the two patient groups, the POAG patients had a significantly higher CDR compared to the PCAG patients (p<0.001).

It has been suggested that the HSPs are involved in the defense mechanism of stress-mediated neuron injury, caused by an increased IOP in glaucoma patients. In addition, these proteins recruit antigen presenting cells, thus they also act as immune stimulatory molecules. Therefore, in addition to their neuroprotective function, when they are overexpressed in the cell, they potentially contribute to disease progression by activating the autostimulatory response that leads to optic nerve neuropathy [17,18]. This is supported by the observation that in glaucoma patients, increased titers of anti-HSP antibodies are present, which facilitate the disease’s progression by diminishing the protective abilities of the native HSPs [33].

This tight regulation of the HSPs’ expression pattern determines the fate of the cell, where underexpression leads to an inefficient stress response and overexpression drives the immunodestruction, with the outcome in both cases being destruction —— driven by the oxidative free radicals in one case and by cytokines in the other, respectively. This is supported by a study conducted by Tezel et al. [33], who have previously shown HSPs to be significant enhancers of both the cytoprotective and neurodegenerative functions of the immune system in RGCs and glial cells, and clearly demonstrated that these proteins were critical in facilitating the glaucomatous changes caused by optic nerve damage and neurodegeneration [33]. Because optic nerve neuropathy in glaucoma is the major cause of blindness in Pakistan, we were therefore interested in studying the possible association between the *HSP70* polymorphism and POAG and PCAG in the Pakistani population.

Our specific interest was to study the stress-mediated responses and their association with glaucoma. We thus chose the G+190C polymorphism for genotypic studies, as this polymorphism has previously been shown to be associated with reduced *HSP70* expression, which could represent an inability to cope with cellular stress and perform cytoprotective functions. Functional analyses of the G+190C polymorphism, which maps to the 5′ UTR region of *HSP70*, have revealed that compared with the G allele, the variant C allele causes reduced promoter activity and lower HSP70 protein levels [34]. Tosaka et al. [19] had previously reported a significant association between the A-110C polymorphism of *HSP70–1* and POAG (p=0.026) in the Japanese population; this polymorphism is known to increase *HSP70* expression, thus supporting the hypothesis that neuropathy is associated with autoimmunity. However, Tosaka et al. [19] did not find any association between glaucoma and the G+190C polymorphism, which supports the alternate model of pathogenicity related to HSP70 cytoprotective functions. As in the Japanese population, we also did not observe any association between the G+190C polymorphism and POAG, but we did find a highly significant association between the polymorphism and PCAG in the Pakistani study group.

One possible manner in which *eNOS* and HSPs might contribute to PCAG is by affecting the expression of matrix metalloproteinases (MMPs). MMPs have been found to be involved in the regulation of IOP and might play a significant role in the normal and pathogenic functions of the eye [35]. The malfunction of the MMP9 protein has previously been shown to be associated with PCAG [36]. The *NOS* gene plays an important role in controlling the activity of MMPs in flow-induced remodeling [37] and hence, the polymorphism we studied could potentially be associated with the overactivation of MMPs, which could then possibly result in PCAG. Other lines of evidence have also suggested a role for *HSP70* in the expression of the *MMP9* gene [38]. It must be pointed out that to fully understand the role of HSPs in the regulation of MMPs, it is important to conduct further expression and activity assays.

In conclusion, glaucoma is a heterogeneous disorder with different environmental and genetic factors driving the etiology of the disease. Various polymorphisms have been reported to be associated with this disease in different populations. Our study represents a step forward in terms of clarifying the role of the stress-related cellular components eNOS and HSP70 and their role in causing glaucoma, particularly in regard to Pakistani patients, and in terms of contributing to the current body of knowledge. There is a further need to conduct studies in different populations across the world to fully unravel the genetic basis of glaucoma, which will ultimately lead to a better understanding of the disease’s underlying mechanisms. Once the disease’s mechanisms are completely understood, better therapeutic interventions can be devised.
